# Technical Innovation for Visual Assessment of Preterm Newborns in a Neonatal Intensive Care Unit: Exploratory Study

**DOI:** 10.1155/2021/9837505

**Published:** 2021-01-04

**Authors:** C. A. Moran, V. L. Alves, S. A. Pereira, M. F. Costa

**Affiliations:** ^1^Department of Health Sciences, Universidade Federal de Santa Catarina, Araranguá, Santa Catarina, Brazil; ^2^Department of Physiotherapy, Faculdade de Ciências Médicas, Santa Casa de São Paulo, São Paulo, SP, Brazil; ^3^Department of Physiotherapy, Universidade Federal do Rio Grande do Norte, Natal, Rio Grande do Norte, Brazil; ^4^Department of Experimental Psychology, Center for Neurosciences and Applied Neurosciences, Institute of Psychology, Universidade de São Paulo, São Paulo, SP, Brazil

## Abstract

**Objective:**

The present study is aimed at assessing heart rate variability (HRV) and its correlation with visual acuity (VA) assessment of preterm newborns (PTNB) in neonatal intensive care units.

**Method:**

Cross-sectional study analyzing HRV during assessment of VA with the aid of a Polar RS800CX heart rate monitor (Polar Electro Oy, Finland). HRV was analyzed according to time and frequency domains and the chaos domain used the autocorrelation coefficient and entropy. The sample consisted of hospitalized PTNB, and static analysis included simple regression diagnosis.

**Results:**

A total of 14 PTNB were included in the sample. VA varied between 0.23 and 1.60 cpd, and only five PTNB obtained below-expected values for age. Statistical analysis demonstrated a negative correlation between VA and time domain (SDDN and SD2) and a positive correlation between frequency domain (heart rate and hertz), but in simple linear regression analysis, these variables did not influence VA.

**Conclusion:**

The results of the study demonstrate that visual acuity was inversely correlated with SDNN and SD2 and during stimulation, showing that the higher the visual performance, the lower the autonomic modulation response.

## 1. Introduction

Autonomic modulation, estimated by heart rate variability (HRV) and regulated by sympathetic and parasympathetic pathways, has been increasingly used as a measure of term and preterm newborn stress and well-being [1–3].

In addition to being considered a sensitive and useful tool to determine autonomic nervous system maturation [4], it also clinically measures the ability to adapt to external events [5], maintain homeostasis [6], and conserve energy [7].

Given the conditions in neonatal intensive care units, preterm newborns are more susceptible to behavioral changes caused by exposure to pain stimuli [8], excessive light and noise [9], and invasive procedures such as mechanical ventilation [10].

When applied to preterm newborns (PTNB), these procedures favor the emergence of complications and comorbidities, including ischemic hypoxic encephalopathy [8], bronchopulmonary dysplasia [11], and visual sequelae such as retinopathy of prematurity [12].

Despite the need for a qualified professional to assess the vision of newborns in neonatal intensive care units, this does not occur in many Brazilian public hospitals and few studies compromising visual development and early PTNB rehabilitation [13, 14].

Thus, we can infer that assessing visual system maturity, using HRV, is an innovative resource, which results in a clear advantage during execution, since it uses heartbeats as a physiological parameter of vagal parasympathetic fiber actions, determining deceleration and sympathetic fiber actions caused by acceleration, because changes in the sensory system of children can be represented by a decrease in the sympathetic system and by the impairment of the parasympathetic system [15].

In addition, studies have demonstrated that organic system immaturity is directly related to gestational age, and ocular structures develop from the gestational period to postbirth [16].

Also, in the context of visual development, the ability to change the focal length of the crystalline lens is controlled nearly entirely by the parasympathetic nervous system [17]. Its parasympathetic excitation contracts the ciliary muscle, which reduces tension, allowing the crystalline lens to become more convex, thereby increasing its dioptric power and enabling the eye to focus on nearby objects [17].

Given that specific visual responses depend directly on the functions of the autonomic nervous system, our question is directly related to the possibility of using a visual marker to identify this neurophysiological function. A possible condition of imbalance could represent an important factor for visual development.

In this respect, the present study is aimed at assessing HRV and its correlation with visual acuity assessment of PTNB in neonatal intensive care units.

## 2. Material and Methods

### 2.1. Study Design and Participants

This is a cross-sectional observational study with 14 PTNB, conducted between January 2016 and December 2017 in the neonatal intensive care units of three public hospitals in São Paulo, Brazil. Participation in the study was voluntary and convenience sampling was obtained from hospital admissions during the study period.

### 2.2. Ethics Committee

The research was approved by the Universidade de São Paulo Research Ethics Committee (protocol no. 53867016.9.0000.5551) and written informed consent was provided by all families who agreed to participate in the study.

### 2.3. Inclusion Criteria

Newborns with gestational age less than 37 weeks, based on the New Ballard Score [18] exhibiting clinical and hemodynamical stability were included. Newborns with variable heart rate or blood pressure, using invasive or noninvasive mechanical ventilation, taking vasoactive or sedative drugs, with grade III or IV periventricular hemorrhage Apgar score in the fifth minute less than 6 congenital malformation, were excluded.

### 2.4. Experimental Procedure

The experimental procedure was carried out in the intermediate care room, with temperature, humidity, and light levels monitored and without significant auditory stimulus.

Data were collected by two specialized researchers, with experience in neonatal intensive care units and qualified to perform visual assessment using Teller acuity cards [19, 20] and operate a heart rate monitor. The procedure was standardized for newborns in the supine position with 30° elevation in a common crib.

Markers were placed on the newborns' chest with Miotec hydrogel and connected to a Polar RS800CX heart rate monitor (Polar Electro Oy, Finland) to assess heart rhythm control.

**Figure 1:**
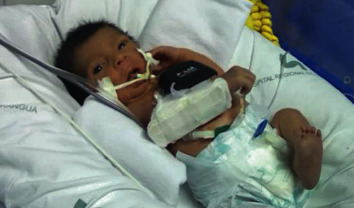
Illustration about the newborn using the heart rate monitor.

The protocol included three measuring times: (1) HRV was assessed initially with the PTNB at rest for 2 minutes to measure baseline heart rate; (2) during the visual acuity assessment protocol for grading with Teller acuity cards II for 6 minutes; and (3) a 2-minute uninterrupted recovery period monitored by the researcher (Figure 1).

### 2.5. Visual Assessment

Grating visual acuity was psychophysically measured using Teller acuity cards II (Vistech Consultants Incorporation). A complete set of Teller Acuity Cards II consists of seventeen 25.5 × 55.5 cm cards, each of which has an approximately 4 mm diameter peephole at the center. Each card is gray with approximately 35% reflectance, varying slightly among cards [16]. The gratings exhibit different spatial frequencies, ranging between 0.23 and 38 cycles/degrees of visual angle, with 1/2 octave spatial frequency between cards [21].

The measure of binocular visual acuity followed the procedure modified by Salomão and Ventura (1995) [22], with the newborns positioned at a standard distance of 55 cm from the stimuli, remaining in the crib throughout the collection period.

The cards were initially presented with gratings of low spatially frequency selected according to age [21]. The task of the experimenter was to test the newborns' reactions to the test and judge whether they looked or not at the grating.

The behavioral reactions observed were the direction of the first gaze, its duration for each stimulus, eye movements, and head movements. The examiner presented both sides of the cards to the newborns. A reaction was considered correct when the child detected the grating on one side of the card and incorrect when it was unable to detect the grating on either side presented.

After each presentation, an assistant informed the examiner whether the reaction was correct or incorrect. Visual acuity thresholds were defined as the average of the values of the last two cards with positive responses and expressed in cycles per degree. Visual acuity classification (normal or reduced) was based on Salomão et al. (2008) [21].

**Figure 2:**
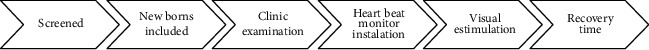
Timeline of the 6 data collection steps.

The timeline with the steps is described in Figure 2.

### 2.6. Assessment of Heart Rate Variability (HRV)

HRV was conducted throughout the collection period and a PTNB heart rate around 120 beats per minute was considered normal, based on the findings of Javorka et al. (2017) [6].

To assess HRV, the data were transmitted to the computer via Polar ProTrainer 5® software. Polar Pro Trainer® software generates a data table of heart rate values. To analyze the data, we used the Kubius® program, a statistical software. The analyses were performed by three exercise zones were classified as rest, assessment, and recovery.

Manual filtering was applied to discard artefacts that could influence the data to be analyzed and only the electrocardiogram data selected were then converted to digital values that reflect cyclical changes in the heart, or the R-R range, and that generate two energy spectra of clinical interest.

For the frequency domain, low frequency (LF), high frequency (HF), and the LF/HF ratio were used.

The first energy spectrum considered was the presence of low frequency wavelengths, standardized in the scientific environment as a power spectrum in the 0.04–0.15 Hz frequency range, which is mediated by the influence of the sympathetic and parasympathetic systems [23].

The second energy spectrum (high frequencies) was also previously established as energy spectra in the 0.15-1.00 Hz frequency range, which are predominantly influenced by parasympathetic activation [23].

The variables analyzed in the time domain were the standard deviation between normal beats (SDNN), the square root of the mean squared difference between adjacent RR intervals (RMSDD), the relationship between standard deviations 1 and 2 obtained by the Poincará-Bendixson graph (SD1/SD2), and the percentage of adjacent intervals with a difference greater than 50 milliseconds (pNN50).

Assessment in the chaos domain used the autocorrelation coefficient (tau) and entropy (ApEn).

### 2.7. Statistical Analysis

The data collected were analyzed using the Statistical Package for the Social Sciences (SPSS)®^,^ version 21.0 (SPSS for Windows, Chicago, USA). The Shapiro-Wilk test was used to determine the normality of the variables. Descriptive statistics was conducted for all the variables and presented as mean and standard deviation. Comparison of heart rates at the different times was analyzed by the ANOVA repeated measures, and additionally, simple regression diagnoses were carried out to determine the predictive ability between the acuity value measured by Teller acuity cards and the significant covariables in bivariate analysis. We considered *p* < 0.05 and 95% CI for all the stages.

## 3. Results

**Figure 3:**
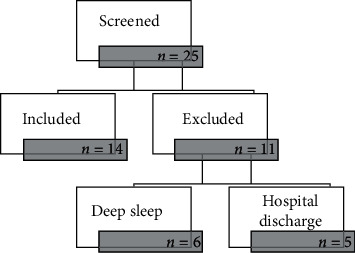
Sample flowchart.

A total of 25 PTNB were screened, 14 of whom were included in the sample, 11 were excluded for being in a deep sleep at the moment of collection (6) or scheduled for discharge before the researchers' visit (5). This sample size has a statistical power of .195 (Figure 3).

Of the 14 PTNB assessed, 7 (50%) were boys with an average chronological age of 7 weeks (±6 weeks). The descriptive data for the sample are presented in Table 1.

VA varied between 0.23 and 1.60 cpd, and only five PTNB obtained below-expected values for age (Tables 2 and 3).

Correlation analysis showed a negative correlation between VA and SDNN (*r* = −0.58, *p* > 0.05), and SD2 (*r* = −0.59, *p* > 0.05), and a positive correlation between HR (*r* = 0.80, *p* > 0.03) and HZ (*r* = 0.80, *p* > 0.03), during Teller assessment, but in simples linear regression analysis, these variables did not influence VA (Table 4).

The RR intervals were performed between 2 : 05 and 14 : 28 minutes (average 8 : 03 minutes), with an overall average of 156 beats (124 to 192 beats).

Repeated measures analysis revealed no difference between the 3 assessment times (before, during, and after) in the time and frequency domains (Table 5).

## 4. Discussion

The results of the study demonstrate that visual acuity was inversely correlated with SDNN and SD2 and during stimulation, showing that the higher the visual performance, the lower the autonomic modulation response. Given this dependence, an inference could be made in both directions, that is, newborns with autonomic dysfunction are at risk of visual impairment, and vice versa. One of the explanations is found in Smith et al. (2013) [5], who suggest that preterm newborns in intensive care units are submitted to several stress stimuli, which, in turn, hinder autonomic nervous system maturation; it can be explained by the possible allostatic load resulting in the impairment of the physiological functioning of several systems of the organism, mainly in the cerebral development [15].

The data show a relationship between visual function and autonomic nervous system modulation and the stress level to which these NBs were submitted. An important contribution of our findings is the fact that we can infer, with a moderate likelihood of success, that visually impaired NBs are more affected by environmental stress. As such, visual measures can be used as a screening tool for risk of physiological stress in this population.

There is enough scientific evidence showing that compromised visual development in preterm [24, 25] and small-for-gestational-age newborns [26] is directly related to the precocity of gestational age. Thus, all preterm newborns are more susceptible to developing visual disorders [27], evident in their poorer visual measures when compared to term newborns [28].

Vision development is a learned skill, which starts at birth in term newborns. The visual pathway from the retina to the cortex is vulnerable during the neonatal period, whereby very low weight newborns are more likely to develop visual disorders such as retinopathy of prematurity [25].

However, although the study included three different intensive care units, only two newborns were visually compromised with retinopathy of prematurity, hindering broader analysis of HRV in NBs with visual disorders.

HRV makes it possible to determine autonomic nervous system maturation and, indirectly, the ability of a NB to adapt to external events. Sympathetic activity is dominant in premature babies, while parasympathetic activity, specifically the vagus nerve, increases with gestational age [3].

Kuerger et al. (2010) [29] compared preterm newborns between 28 and 34 weeks of age with their term counterparts and found that the autonomic nervous system in this period exhibited maturation, particularly of the parasympathetic pathway [29], in line with the profile of the present sample, which was evaluated at a corrected age of 40 weeks. Newborns with gestational age of more than 36 weeks demonstrated significantly greater HRV than their younger counterparts [6].

Although studies showed sympathetic pathway activity in the preterm newborns of the present study, it was predominantly parasympathetic, which may be explained by visual acuity stimulation from the Teller acuity cards, revealing autonomic maturation at a frequency above 0.15 hertz, confirmed by the positive correlation, represented by the increase in HR and HZ, and good visual performance of premature infants.

However, we found a negative correlation between SDNN and SD2, showing that the better the visual acuity, the lower the autonomic system activation. SDNN represents a balance between the sympathetic and parasympathetic nervous systems, whereas SD2 is equivalent to activation of the sympathetic nervous system.

Average SDNN in the present study was 13 ms, similar to that reported by Selig et al. 2011 [7], in preterm infants. This finding may be due to the fact that only a single assessment was carried out in the first days of life, with no monitoring of neurological maturation, and the significant difference in the visual system.

Moreover, the lower vagal response, represented by the sympathetic system, is directly related to morbidities, which influence neurotransmitter release into the central nervous system, since the sympathetic nervous system is responsible for releasing energy as well as participating in glucose and fat metabolization [30]. The fact that our sample consisted of stable newborns, with definitive diagnosis and average weight of 2.584 kg, suggests a state of physiological stability and well-being at assessment.

Although gender-related HRV factors are already present at birth, the literature findings remain controversial [6]. However, in the present study, no significant gender differences were found.

Visual assessment of newborns in intensive care units is routinely conducted by neonatologists via red reflex, the procedure recommended by the American Academy of Pediatrics, aimed at early detecting of visual disorders and carried out as a protocol to identify eye diseases [31]. However, measuring visual acuity by Teller cards is a rapid clinical method, validated for the Brazilian infants population [22], and enables estimating visual acuity by the spatial frequency of the best grating, which can be observed in the present study.

Early detection of visual disorders and effective treatment in rehabilitation programs results in better motor and cognitive performance and social inclusion of the child [32], making it essential to implement low-cost, easy-to-apply tools.

### 4.1. Study Limitation

The main study limitation was recruiting the sample in neonatal intensive care units, due to the severity of the clinical cases and the transfer of several newborns to other wards in the hospital. Nevertheless, the tool is easy to apply and noninvasive, thereby enabling better understanding of preterm newborn development.

In addition, another study limitation is the lack of evidence to confirm biomarkers of HRV as inducers of autonomic nervous system responses in newborns, given the complex nonlinear interactions between the sympathetic and parasympathetic pathways [33, 34].

Thus, the visual assessment can complement analysis of autonomic nervous system maturation. In addition, physiological biomarkers can complement visual tests and be used to noninvasively measure behavioral visual acuity and track NBs at risk of impaired nervous system development.

During VA assessment, PTNB had a predominantly parasympathetic activity, which may be explained by stimulation from the Teller acuity cards. The results suggest an ability of the crystalline lens to increase its dioptric power and enabling the eye to focus on Teller acuity cards, but further studies need to confirm this hypothesis.

## 5. Conclusion

The results of the study demonstrate that visual acuity was inversely correlated with SDNN and SD2 and during stimulation, showing that the higher the visual performance, the lower the autonomic modulation response.

## Figures and Tables

**Table 1:** Descriptive characteristics of the sample.

Variable	Mean (SD)
Gestational age (weeks)	(32 ± 2.17)
Birth weight (kg)	1,619.00 (±770.21)
Current weight (kg)	2,584.08 (±952.63)
VA (cpd)	0.877 (±0.528)

cpd: cycles per degree; kg: kilograms; SD: standard deviation; VA: visual assessment.

**Table 2:** Individual sample values.

Identification	Sex	Apgar in the first minute	Apgar in the fifth minute	Gestational age (weeks)	Days of life	Birth weight (g)	Resting HR (BPM)	HR during Teller (BPM)	Recovery HR (BPM)
1	F	4	7	29	785	3460	179	180	178
2	M	7	9	30	980	2015	122	124	128
3	F	7	9	30	940	1875	144	146	145
4	M	6	7	37 6/7	3410	3255	141	157	168
5	F	7	8	32	1595	1930	171	165	166
6	F	7	9	33 4/7	2185	2150	138	175	153
7	F	7	9	32 4/7	1400	2580	146	141	128
8	F	9	10	35	1970	1975	160	165	160
9	M	8	9	32 5/7	1760	1970	142	137	146
10	F	7	8	34	1905	2354	160	161	166
11	M	7	9	30	1350	2320	166	167	167
12	M	4	7	36 6/7	3620	3620	124	132	130
13	M	1	5	31 5/7	1015	1955	184	192	188
14	M	7	9	33 6/7	2115	4955	146	149	156

**Table 3:** Continuation of individual sample values.

Identification	Resting (Hz)	Hz during Teller	Recovery Hz	Assessment with Teller (cpd)	Initial reference value (cpd)	Reference value (average-cpd)	Reference value (lower-cpd)	Final result
1	2.98	2.3	2.97	1.3	0.44	3.89	1.49	0
2	2.04	2.06	2.13	1.6	0.44	3.89	1.49	0
3	2.4	2.44	2.41	0.43	0.44	3.89	1.49	0
4	2.35	2.62	2.79	0.23	0.3	0.66	0.27	0
5	2.86	2.75	2.83	0.43	0.3	0.66	0.27	0
6	2.3	2.92	2.55	0.23	0.3	0.66	0.27	0
7	2.44	2.36	2.14	0.86	0.3	0.66	0.27	1
8	2.67	2.76	2.67	0.86	0.3	0.66	0.27	1
9	2.36	2.29	2.43	1.6	0.3	0.66	0.27	1
10	2.66	2.68	2.76	0.23	0.3	0.66	0.27	0
11	2.77	2.85	2.79	0.32	0.3	2.02	0.26	0
12	2.07	2.19	2.17	1.6	0.3	0.66	0.27	1
13	3.07	3.2	3.13	0.86	0.3	0.66	0.27	1
14	2.44	2.49	2.6	0.43	0.3	0.66	0.27	0

BPM: beats per minute; cpg: cycles per degree; cpd: cycles per degree; F: female; M: male; HR: heart rate; Hz: hertz; min: minute; VA: visual acuity value; initial, average, and lower reference value: normative VA values reported by Salomão, 1995 [22]; 0: visual acuity expected for age; 1: altered visual acuity.

**Table 4:** Simple linear regression analysis between visual acuity and different HRV domains.

Model	*β*	*p*	95% confidence interval	
			Lower CI	Upper CI
HR	10.577	.395	-.289	.648
HZ	-9.871	.425	-37.965	17.941
SDNN	-.175	.508	-.048	.026
SD2	. 491	.884	-367	.415

Dependent variable: visual acuity; independent: frequency domain variables (HR: heart rate and HZ: hertz) and time domain variables (SDNN: standard deviation between normal beats; SD2: standard deviation) during test; *β*: beta; *p*: *p* value. Linear regression analysis.

**Table 5:** Repeated measures analysis.

Variables	Rest	Assessment	Recovery	*p*
HR	151 ± 5.0	152 ± 5.2	158 ± 5.3	0.29
HZ	2.53 ± 0.28	2.54 ± 0.28	2.64 ± 0.29	0.24
LF (ms^2^)	0.07 ± 0.01	0.07 ± 0.01	0.06 ± 0.01	0.42
HF (ms^2^)	0.18 ± 0.06	0.18 ± 0.06	0.16 ± 0.01	0.51
LF/HR	0.42 ± 0.13	0.44 ± 0.16	0.38 ± 0.22	0.77
SDNN (ms)	13.85 ± 8.82	12.60 ± 4.64	13.78 ± 7.43	0.72
RMSSD (ms)	9.98 ± 9.84	7.79 ± 4.55	11.06 ± 9.29	0.21
PNN50	0.76 ± 1.41	0.36 ± 0.63	0.42 ± 0.52	0.75
SD1	6.18 ± 4.59	5.50 ± 3.23	7.80 ± 6.58	0.24
SD2	18.57 ± 11.68	16.92 ± 5.90	17.66 ± 8.67	0.88
SD1/SD2	0.31 ± 0.85	0.31 ± 0.93	0.41 ± 0.15	0.1
APEN	0.80 ± 0.29	0.77 ± 0.29	0.81 ± 0.38	0.95

ANOVA repeated measures; ApEn: entropy; HR: heart rate; Hz: hertz; HF: high frequency; LF: low frequency; ms: milliseconds; PNN50: percentage of differences between adjacent normal RR intervals greater than 50 milliseconds; RMSSD: root mean square of the successive differences between adjacent RR intervals; SDNN: standard deviation between normal beats; SD: standard deviation.

## Data Availability

The data base is available with author no. 1 and could be asked in anytime by e-mail.

## Additional Points

*Official Institution to Which the Work is Linked*. Federal University of the Santa Catarina.
